# Integrating ecological approaches to interrupt schistosomiasis transmission: opportunities and challenges

**DOI:** 10.1186/s40249-018-0506-4

**Published:** 2018-12-12

**Authors:** Song Liang, Eniola Michael Abe, Xiao-Nong Zhou

**Affiliations:** 1 Department of Environmental and Global Health, College of Public Health and Health Professions, and Emerging Pathogens Institute, University of Florida, Gainesville, FL 32610 USA; 2 National Institute of Parasitic Diseases, Chinese Center for Disease Control and Prevention; Key Laboratory of Parasite and Vector Biology, Ministry of Health, Shanghai, 200025 China; 3 National Center for International Research on Tropical Diseases, Ministry of Science and Technology; Chinese Center for Tropical Diseases Research; WHO Collaborating Centre for Tropical Diseases, Shanghai, 200025 China

**Keywords:** Schistosomiasis, Transmission interruption, Ecological framework, Integrated control

## Abstract

**Background:**

The development of agenda for global schistosomiasis elimination as a public health problem generates enthusiasms among global health communities, motivating great interests in both research and practice. Recent China-Africa schistosomiasis control initiatives, aiming to enhance collaboration on disease control in African countries, reflect in part that momentum. Yet there is a pressing need to know whether the Chinese experiences can be translated and applied in African settings.

**Main body:**

China’s remarkable achievements in schistosomiasis control programme, associated experiences and lessons, have much to offer to those combating the disease. Central to the success of China’s control programmes is a strategy termed “integrated control” – integrating environmental approaches (e.g. improved sanitation, agricultural and hydrological development and management), which target different phases of the parasite transmission system, to chemical-based drug treatment and mollusciciding. Yet, despite significant measurable public health benefits, such integration is usually based on field experience and remains largely uncharacterized in an ecological context. This has limited our knowledge on relative contributions of varying components of the integrated control programme to the suppression of disease transmission, making it challenging to generalize the strategy elsewhere. In this opinion article, we have described and discussed these challenges, along with opportunities and research needs to move forward.

**Conclusions:**

There is an urgent need to formalize an ecological framework for the integrated control programme that would allow research towards improved mechanistic understanding, quantification, and prediction of the control efforts.

**Electronic supplementary material:**

The online version of this article (10.1186/s40249-018-0506-4) contains supplementary material, which is available to authorized users.

## Multilingual abstracts

Please see Additional file 1 for translations of the abstract into six official working languages of the United Nations.

## Background

At the 2018 Forum on China-Africa Cooperation (FOCAC) summit held in Beijing on September 3, 2018, the Beijing Declaration, *Toward an Even Stronger China-Africa Community with a Shared Future* and *the FOCAC Beijing Action Plan* (2019–2021) were stipulated, in which China-Africa cooperation targeting important public health issues, including schistosomiasis, has been prioritized. This has added further momentum to recent China-Africa initiatives on schistosomiasis control.

Schistosomiasis is an important neglected tropical disease (NTD), which remains a serious public health problem in the tropics and subtropics with more than 250 million people infected in 78 countries [1]. Yet, the world has witnessed dramatic reductions in disease burdens associated with *Schistosoma* spp. infections in many endemic areas in the past few decades. Importantly, successful control programmes and even focal elimination of transmission have been achieved in some endemic countries in the Americas, North Africa, the Middle East, and Asia. Such success, together with the needs of global sustainable development, has motivated the development of an agenda for schistosomiasis elimination at global scale. The 65th World Health Assembly in 2012 set the goal of archiving schistosomiasis elimination in the Americas and Western Pacific Regions, potentially eliminating the disease as a public health problem in multiple countries in Africa by 2020, and finally achieving the global elimination of schistosomiasis as a public health problem by 2025 [2].

This is exciting for global health and development, particularly for sub-Saharan Africa (SSA) where ~ 93% of current schistosomiasis burden resides [3]. The key question facing global health communities is how to achieve the goals in the years to come. This is not an easy question to answer, but looking back at what happened in history, especially the achievements reported in countries where such goals had already been achieved or are close to being achieved, might help to shed light on the game plans.

Strategically, mass drug administration (MDA) has been the mainstay of global schistosomiasis control strategies and, encouragingly, has resulted in significant measurable health gains [4]. Yet, with mounting evidence from studies on all *Schistosoma* spp. of major public health concerns, it becomes clear that MDA alone is insufficient to achieve widely sustainable elimination of schistosome transmission [5–8]. In some endemic countries, a combination of MDA with control of snail intermediate hosts, for instance, via chemical-based mollusciciding, has resulted in more sustainable control effects [9, 10], offering evidence on the importance of coupling other strategies with drug treatment-based strategy.

China’s overall success in controlling schistosomiasis brings a particular excitement from this perspective. The country had > 10 million infected people in 12 endemic provinces in the 1950s and brought down to less than 10 000 in five provinces in 2017 [11]. As of now, the overall prevalence of infection is < 1% across endemic areas and the disease’s public health burden has been significantly reduced. In 2018, Sichuan Province, once a severely endemic area, became the latest province that also declared achieving the goal of province-wide transmission interruption of schistosomiasis.

So what strategies are underlying the success of control programmes in China? While a variety of approaches were explored in the past, central to the success has been a strategy called “integrated control” adopted as the key strategy of the national programme. The strategy emphasizes sustainability and integration of interventions from the public health sector - infectious source control and case treatment – with environmental management programmes typically sponsored by other sectors such as agricultural and hydrological departments, for instance, from infection source management (e.g. human waste and livestock) to agricultural development (e.g. crop structure shifting and irrigation system development less amendable to the disease transmission). Such strategy has yielded both considerable public health and socio-economic gains.

**Fig. 1:**
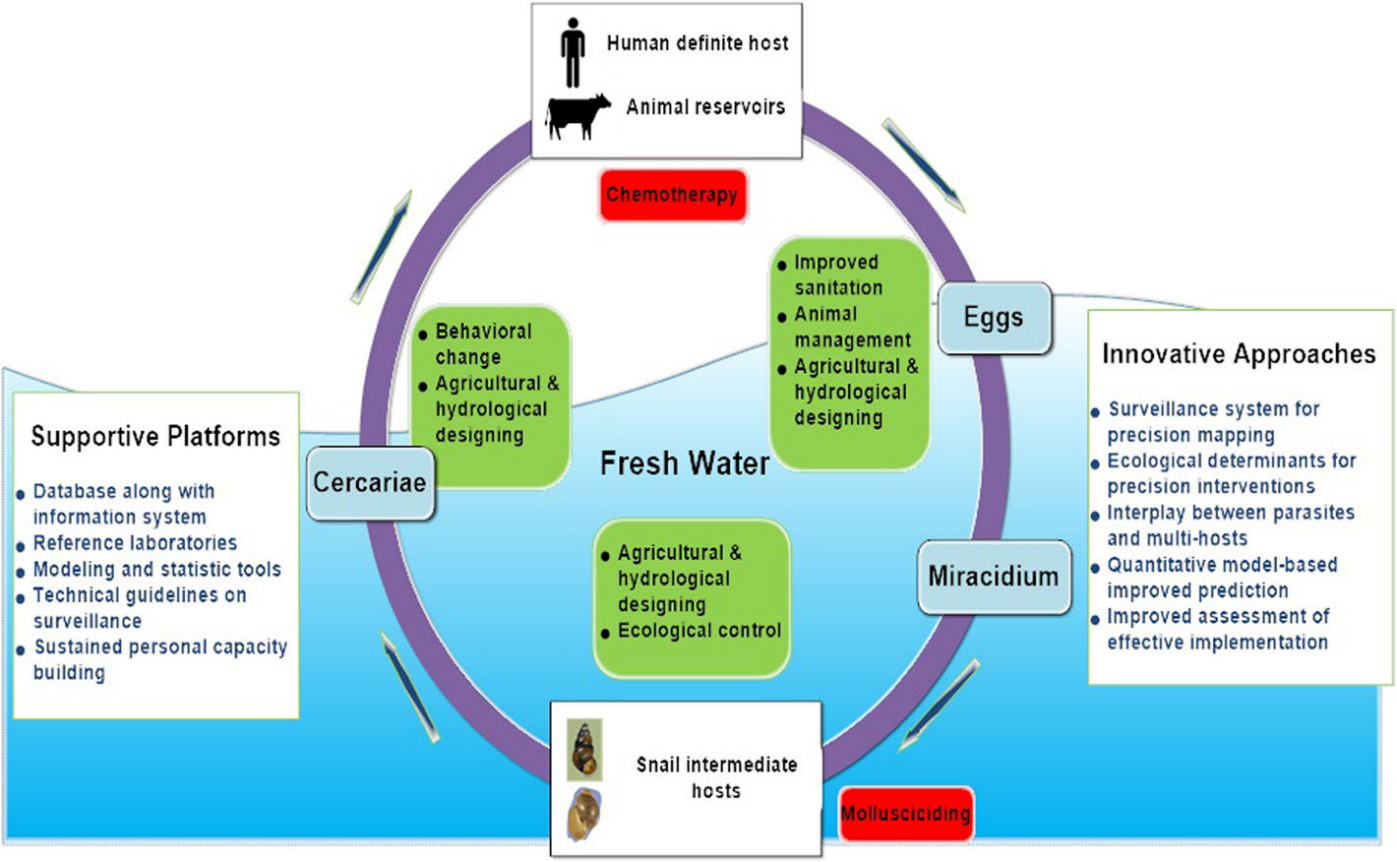
Integrated interventions in the ecological framework. Integrated interventions, targeting various aspects of parasite transmission, primarily consist of *infectious source control* (e.g. chemotherapy on infected humans and/or animals, animal and waste management through improved sanitation), *control of snail intermediate host* (e.g. mollusciciding using niclosamide, agricultural and hydrological management, and afforestation), and *exposure control* (e.g. behavioral change through hygiene improvement, and agricultural and hydrological management). However, the relative contribution of integrated intervention framework to the suppression of schistosomiasis remain largely uncharacterized both mechanistically and quantitatively. This limits the generalizability of these intervention approaches to endemic areas elsewhere. The ecological framework emphasizes dynamic interactions among different system components as illustrated in the figure. Such framework can allow the potential usage of different tools to quantify these interventions and associated impacts. The commonly used approach is dynamic modeling, in which these interventions can be specifically factored. The widely used metric for transmission of infectious diseases, R_e_, effective reproductive number (or transmission potential) may be used to assess the impacts of the integrated interventions on disease transmission and efforts needed to eliminate the transmission. While ecology-based intervention including environmental modification through water resources projects or agricultural projects will be the major components to provide long-term effectiveness and precision intervention towards schistosomiasis transmission interruption. Biological and mechanical control methods such as application of extracted plant molluscicides and introduction of natural predators (e.g. snail eating fishes or prawns) of intermediate hosts can be harnessed to effectively control snails

Is the integrated control generalizable to elsewhere? This question is especially relevant given the recent China-Africa initiatives for joint programmes against schistosomiasis in SSA. In China, the integrated control has been largely guided by some practical experiences from various field trials (Fig. 1). However, a lot of questions remain unanswered. For example, what is the relative contribution of improved sanitation versus agricultural development to the suppression of schistosomiasis transmission in a specific environmental setting? What is the optimal combination of drug treatment and environmental management/modification to maximize cost-effectiveness of disease control? Improved knowledge about how the integrated control works will offer insights into generalizability and feasibility of such control strategies while working in different settings. To archive this, a formal ecological framework is needed for conceptualizing, quantifying, and predicting effects of these control strategies, and validating them before applying to a different setting. Here we present some thoughts on both challenges and opportunities moving forward along with the research needs.

## Main text

### Challenges

A schistosomiasis control programme, particularly when towards elimination, requires long-term sustainable support, both financially and technically, from multi-sectoral commitments to transdisciplinary collaborations [4]. Hence, the following challenges are anticipated when proposing integrated control in the ecological framework.

First, with the decline in disease prevalence, governments may lose drive to continue making investment on control involving environmental management and/or modifications. This might lead to unsustained integrated efforts of the control programmes and consequently, unabated transmission or resurgence of schistosomiasis transmission, particularly in lake region of China. Keeping the governments’ long-term engagement, from initial control to elimination, is essential for sustainability of the integrated control programme. Fortunately, China’s central government is committed to such long-term support and a recent call for the three-year national action plan to sustain the multi-sectoral cooperation has further laid foundation toward the goal of nation-wide schistosomiasis elimination.

Second, despite the great success of national schistosomiasis control programmes through the implementation of integrated control strategy, some operation challenges emerge, for instance, developing cost-effective ecological intervention packages that target specific transmission environments. Ecological interventions usually involve multiple sectors and are costly, requiring close coordination among different stakeholders to maximize public health gains. Enhancing cost-effectiveness is one of critical elements for sustainability of the control programmes. Innovative ideas and tools are much needed moving forward along the path of integrated control in the elimination stage [12].

Third, ecological intervention (e.g. environmental management/modification) requires participation of local rural communities, in particular young population. Yet, rapid rural development and urbanization have prompted migration of the majority of young people to urban settings, making the control implementation more challenging due to diminishing participation of young population. Furthermore, rural residents are examined and treated by local health professionals, but these young migrant workers often go undiagnosed and untreated in their urban homes, leading to morbidity and raising the risk of reintroduction of disease into previously controlled rural areas when these workers return home to visit.

Finally, weak health systems and lack of resources in most endemic countries of Africa will pose enormous challenges to broadly apply ecological interventions to control schistosomiasis and other NTDs as well as contribute to high disease burden [13].

### Opportunities

The integrated control, typically involving environmental management and modifications with strong ecological focus, has proven effective in China [14]. Using this strategy, China has achieved remarkable success in the sustainable schistosomiasis control across the country [15]. The companioned improvements in access to drinking water and sanitary facilities through the integrated control [16] have also generated other public health benefits, for instance, reducing diarrhea incidence and other neglected tropical diseases (e.g. soil-transmitted helminth infections) [6].

In addition to the public health gains, equally significant is the agricultural benefit (e.g. increase in profit through agricultural yield or shifting agriculture) in rural environment through the integrated approach. The public health and agriculture co-benefits provide great incentives for participation of local residents in the control programmes [17].

The recent China-Africa Initiatives for schistosomiasis control provide unprecedented opportunities for the disease control programmes in Africa. Workshops and training courses designed for African counterparts, as well as mutual field visits have facilitated exchange of ideas and control experiences etc. With the support of Chinese central government, collaborations on schistosomiasis control between China and Africa are expected to deepen and expand in the years to come [13].

### Research needs

**Fig. 2:**
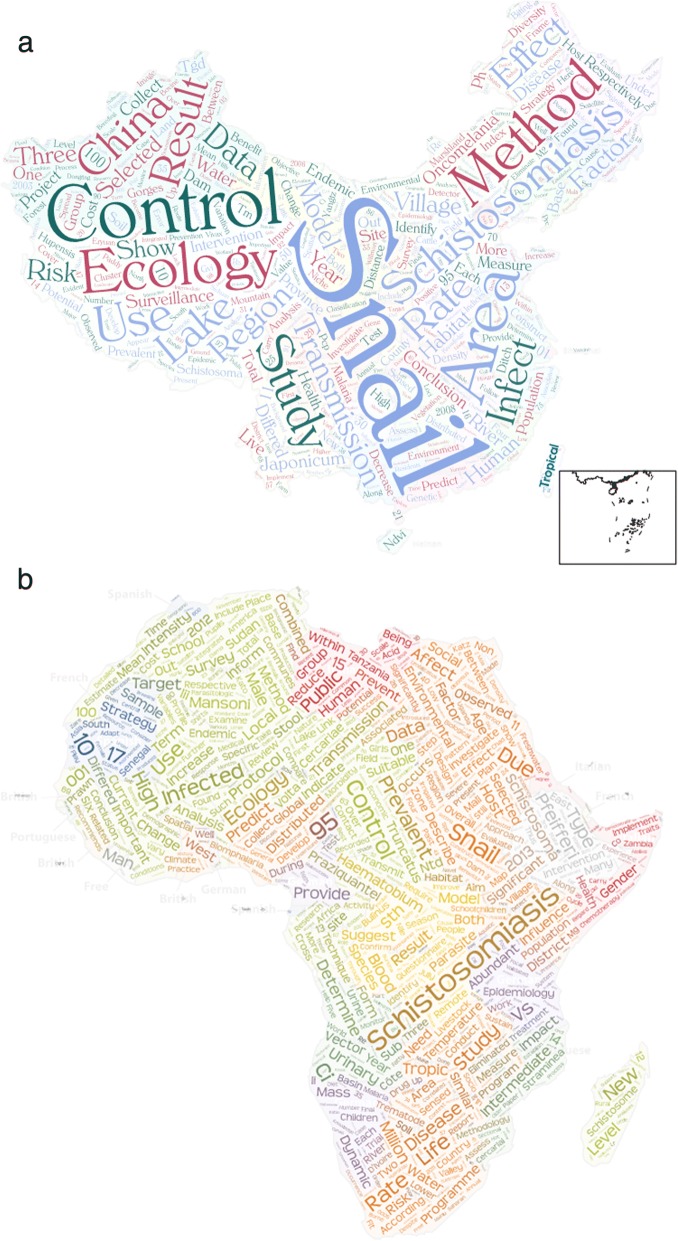
The WordCloud maps of ecological intervention applied in the national schistosomiasis control programmes in China (**a**) and Africa (**b**). Two WordClouds were generated in R using the following steps: (1) The search terms of (“ecological intervention” AND “schistosomiasis intervention” AND “China/Africa”) were used to search PubMed database (2)Searched results including titles and abstracts in all literatures of PubMed database, were loaded into tidytext, dplyr, stringr and wordcloud2 packages in R solfware (version 3.5.1), and finally produced interactive wordclouds, with the assistance of China and Africa contour maps.

Following the initiation of schistosomiasis elimination programme at the national level as proposed by the World Health Organization, surveillance has been prioritized at the elimination stage in each endemic country. Technically, these initiatives need to be supported by two pillars – development of precision maps for decision makers and ecological intervention for sustainable and cost-effective benefits. However, there is a pressing need to close critical knowledge gaps on ecological interventions through further research, based on the gap analysis between China and Africa regions which illustrated in Fig. 2.

First, there is a need to improve and update surveillance mechanisms for sustainable ecological monitoring and evaluation. This can be achieved using important precision mapping tools to provide smooth and predictive risk maps on snail hosts habitat preference [18]. Identifying and integrating different biologic and ecologic factors responsible for snail hosts choice of habitat preference can inform decision making and control implementation.

Second, the need to improve our understanding on ecological determinants of *Schistosoma* spp. transmission across socio-environmental landscapes, including human behaviors, environmental changes, economic development, biotic and abiotic factors relevant to schistosomiasis transmission. Particularly, global changes (e.g. environmental and anthropogenic) have promoted infectious diseases transmission and are presumably responsible for changes in species geographical distribution and hybridization [19]. Novel mathematical or statistical models provide a powerful tool that would improve our understanding about the complex interplay leading to the establishment of new foci and also quantify forces of infection.

Third, the intricate interplay between the environment, parasites and hosts provides opportunities for genetic exchange among parasites through the hosts leading to possible development of new strains, and these pose serious challenges to the control programmes [19]. Improvement in the application of immunomics and next-generation sequencing platforms has provided great opportunities for research of this nature to broaden our understanding on cross-species transmission of the pathogen [20].

Fourth, there is need to develop operational research on evaluation of cost-effectiveness under the comprehensive environmental accounting frameworks, for instance, develop and/or update models to determine best cost-effectiveness and cost-benefit approaches within the ecological intervention framework [21]. This will guide policy makers to develop policies and monetary guidelines for control implementation, and can also help transfer successful ecological intervention model to other areas where needed.

## Conclusions

MDA using praziquantel to control schistosomiasis morbidity has created some debates on possibility of reducing schistosomiasis burden in the targeted groups. However, studies have shown that MDA alone will not be sufficient to achieve Sustainable Development Goals related to schistosomiasis elimination targets by 2030. Meanwhile, building on the progress achieved with MDA treatment, interventions by implementing ecological control strategies would effectively interrupt schistosomiasis transmission in many endemic foci.

China has implemented the integrated control strategy since the strategic review of their schistosomiasis control plan in 2004 and has successfully reduced schistosomiasis transmission to < 1% prevalence across endemic areas of the country [15]. This achievement is laudable and it is, therefore, imperative that such experiences be transferred and adapted to other schistosomiasis endemic foci especially in SSA which account for the highest disease burden.

With the Chinese government increasing interest in global health agenda and their commitment to support African countries to curtail schistosomiasis transmission, the institution-based network on China-Africa cooperation for schistosomiasis elimination (INCAS) has been established to enhance capacity building, knowledge transfer and integration of ideas towards blocking schistosomiasis transmission in Africa. The Chinese experts will use this platform to distil their experiences as well as assess the feasibility and effectiveness of the ecological control strategies when adapted to different epidemiological settings across Africa.

## Additional file

Additional file 1: Multilingual abstract in the six official working languages of the United Nations. (PDF 602 kb)

## Abbreviations

FOCAC: Forum on China-Africa Cooperation
INCAS: Institution-based network on 295 China-Africa cooperation for schistosomiasis elimination
MDA: Mass drug administration
NTD: Neglected tropical disease
SSA: Sub-Saharan Africa
