# Supplementary material for: Integrating ecological approaches to interrupt schistosomiasis transmission: opportunities and challenges
Source: Infect Dis Poverty. 2018 Dec 12;7:124. doi: 10.1186/s40249-018-0506-4 (PMC6291957; doi:10.1186/s40249-018-0506-4)
Supplement: Supplementary file 1 — Multilingual abstract in the six official working languages of the United Nations. (PDF 602 kb) [file 40249_2018_506_MOESM1_ESM.pdf]

Translation of the abstract into the six official working languages of the United Nations

دمج كامل الحلول البيئية لإيقاف انتشار البلهارسيا: الفرص والتحديات

سونغ ليانغ، إنيولا مايكل أيب، شياو-نونغ جو

الملخص
الخلفية: يولد تطور برامج القضاء على البلهارسيا عالمياً كمشكلة صحة عامة، الحماس بين جماعات الصحة العالمية، دافعاً لاهتمام أكبر بالبحث والتطبيق معاً. إن المبادرات الحديثة لمكافحة بلهارسيا الصين وإفريقيا، والتي تسعى لتعزيز المشاركة في مكافحة المرض في الدول الإفريقية، تعكس في جزء منها هذا العزم. ومع ذلك هنالك حاجة ملحة لمعرفة إذا كان من الممكن نقل وتطبيق التجارب الصينية على الظروف الإفريقية.
النص الرئيسي: لدى إنجازات الصين البارزة في برنامج مكافحة البلهارسيا، والتجارب والدروس المرافقة، الكثير لإعطاءه لأولئك الذين يكافحون المرض. الجزء الأساسي لنجاح برنامج المكافحة في الصين هي استراتيجية تدعى "المكافحة المتكاملة" -أي دمج كامل الحلول البيئية (كتحسين الصرف الصحي، والتطوير والتنظيم الزراعي والمائي)، التي تستهدف مراحل أخرى من نظام انتقال الطفيلي، إلى العلاج الدوائي الكيميائي ومبيدات الحلزونات. مع هذا وبالرغم من الفوائد البارزة على الصحة العامة التي يمكن قياسها، فإن دمجاً كهذا عادةً ما يكون مرتكزاً على التجربة الميدانية ويبقى لحد كبير غير مميز في إطار بيئي. حد هذا من معرفتنا للإسهامات المؤثرة في المقومات المختلفة لبرنامج المكافحة المتكاملة من أجل إعاقة انتشار المرض، مما صعّب تعميم الاستراتيجية في مكان آخر. ففي مقال الرأي هذا، شرحنا وناقشنا هذه التحديات، بالإضافة إلى الفرص واحتياجات البحث للتقدم للأمام.
الاستنتاجات: هناك حاجة ملحّة لصياغة هيكل عمل بيئي لبرنامج المكافحة المتكاملة، مما سيفتح المجال أمام البحث نحوتحسين الفهم الآلي، وقياس، وتوقع جهود المكافحة.

Translated from English version into Arabic by Nirmin Hashoum, proofread by Kowthar Alasady, through

阻断血吸虫病传播的综合性生态学方法：机遇和挑战

梁松, Eniola Michael Abe, 周晓农

摘要
引言：全球消除血吸虫病公共卫生危害的规划制定已对全球卫生领域产生了巨大影响，也促进了该领域的研究与防控工作。这方面的进展，也包括了旨在加强在非洲国家的血吸虫病防控工作中非血吸虫病合作项目的启动。的确，如何将中国经验移植并能适用于非洲情况，是一项急需解决的工作。
主要内容：中国在血吸虫病防治工作中取得了巨大成绩，能为其它地区开展疾病防控工作提供有价值的经验与教训。中国血吸虫病防治工作的成功经验在于“综合防治”策略，这包括针对寄生虫病传播环节中不同阶段的环境治理方法（如改水改厕、农业与水利开发工程与管理等）与化学药物治理和灭螺工作的综合。尽管这些工作取得了巨大的公共卫生效益，这些工作通常借助于现场防治实践经验，但尚少对生态学概念进行定义与刻画。这导致了我们对综合防控规划中不同方面对疾病传播作用的认知局限性，从而难以提出可推广的防控策略。我们在本文中重点描述了这些工作中的挑战，以及今后工作的机遇和研究需求。

结论：基于提升疾病防控机制、定量指标和预警等方面的研究，从而制定一个疾病综合防控规划的生态学框架已迫在眉睫。

Translated from English version into Chinese by Xiao-Nong Zhou

Intégration d’approches écologiques afin d’arrêter la transmission de la schistosomiase: opportunités et défis

Song Liang, Eniola Michael Abe, Xiao-Nong Zhou

Résumé

Contexte général: l’élaboration d’un programme visant à l’éradication dans le monde entier de la schistosomiase qui constitue un problème de santé publique suscite l’enthousiasme des communautés sanitaires internationales et éveille un grand intérêt tant à l’égard de la recherche que de la pratique. Les récentes initiatives sino-africaines de lutte contre la schistosomiase, qui visent à renforcer la collaboration en matière de lutte contre la maladie dans les pays africains, reflètent en partie cet intérêt. Il est pourtant urgent de savoir si les expériences chinoises peuvent être mises en pratique et utilisées dans un contexte africain.

Structure principale: les réalisations remarquables de la Chine dans le cadre du programme de lutte contre la schistosomiase, les expériences et les enseignements qui en découlent constituent de précieux éléments dans la lutte contre cette maladie. Le succès des programmes chinois de lutte repose sur une stratégie appelée « lutte intégrée » qui intègre des approches environnementales (p. ex. amélioration de l’assainissement, développement et gestion de l’agriculture et de l’hydrologie), qui ciblent différentes phases du système de transmission du parasite, le traitement médicamenteux à base de substances chimiques et le recours à des molluscicides. Pourtant, en dépit d’importants avantages mesurables pour la santé publique, une telle intégration est habituellement fondée sur l’expérience sur le terrain et demeure largement imperceptible dans un contexte écologique. Cette contrainte a limité nos connaissances des contributions relatives de chaque composante du programme de lutte contre la transmission des maladies, ce qui complique la généralisation de la stratégie dans un autre contexte. Dans le présent article argumentatif, nous avons décrit et abordé de ces défis, ainsi que les opportunités et les besoins de recherche afin de progresser.

Conclusions: il est urgent de formaliser un cadre écologique pour le programme de lutte intégrée qui permettrait d’effectuer des recherches visant à améliorer la compréhension du mécanisme, la quantification et la prévision des efforts de lutte.

Translated from English version into French by Gabriela Kouahla, proofread by Eric Ragu, through

TRANSLATORS
WITHOUT BORDERS

Интеграция экологических подходов к прекращению передачи шистосомоза: возможности и трудности

Сун Лян, Эниола Майкл Абе, Сяо-Нун Чжоу

Аннотация

Краткое описание: Разработка плана действий по всеобщей ликвидации шистосомоза как проблемы общественного здравоохранения была с энтузиазмом встречена глобальными сообществами по охране здоровья, стимулируя огромный интерес к научным исследованиям и практике. Недавние совместные инициативы Китая и Африки по борьбе с шистосомозом, направленные на расширение сотрудничества в области контроля заболеваемости в африканских странах, частично отражают динамику этого движения. Тем не менее, существует острая необходимость понимания возможности адаптации и применения китайского опыта в африканских условиях.

Основная часть: Впечатляющие достижения программы Китая по борьбе с шистосомозом, приобретенный в ходе программы опыт и извлеченные уроки чрезвычайно полезны тем, кто пытается преодолеть эту болезнь. Главной причиной успеха программ Китая по борьбе с заболеванием является стратегия под названием «интегрированный контроль», которая заключается в интеграции экологических подходов (например, улучшенные санитарные меры, сельскохозяйственное и гидрологическое развитие и управление), нацеленных на разные фазы системы передачи паразита, а также лекарственное лечение на основе химических препаратов и уничтожение моллюсков. Однако несмотря на значительную измеримую пользу для общественного здоровья, такая интеграция обычно основана на практическом опыте на местах и в значительной степени остаётся не охарактеризованной в экологическом контексте. Это ограничило наши знания об относительном вкладе изменяющихся компонентов программы интегрированного контроля по ликвидации передачи болезни, что затрудняет обобщение стратегии её применения в других регионах. В данной статье-мнении мы описали и обсудили указанные трудности, а также возможности и исследования, необходимые для продвижения вперед.

Выводы: Необходимо в безотлагательном порядке формализовать экологическую модель для интегрированной программы контроля, которая бы позволила проводить научные работы, направленные на улучшение механистического понимания, количественной оценки и прогнозирования усилий по борьбе с болезнью.

Translated from English version into Russian by Natalia Potashnik, proofread by Liudmila Tomanek, through

TRANSLATORS
WITHOUT BORDERS

Integrar propuestas ecologistas para interrumpir la transmisión de la esquistosomiasis: oportunidades y desafíos

Song Liang, Eniola Michael Abe, Xiao-Nong Zhou

Resumen

Antecedentes: El desarrollo de un programa para erradicar la esquistosomiasis a nivel mundial como un problema de salud pública entusiasma a las comunidades sanitarias de todo el mundo y genera un gran interés tanto en la investigación como en la práctica. Las recientes iniciativas chino-africanas para luchar contra la esquistosomiasis, que pretenden impulsar la colaboración para combatir la enfermedad en los países africanos, reflejan en parte este ímpetu. Sin embargo, urge saber si las experiencias chinas pueden extrapolarse y aplicarse en el contexto africano.
Cuerpo: Los grandes logros de China en su lucha contra la esquistosomiasis, junto a las experiencias y enseñanzas que ha obtenido, son de gran valor para aquellos que combaten la enfermedad. El éxito chino se debe principalmente a una estrategia llamada "control integral", que combina propuestas medioambientales (p. ej. mejorar el saneamiento y el desarrollo y la gestión agrícola e hidrológica) dirigidas tanto a las distintas fases del sistema de transmisión del parásito como al tratamiento farmacológico y los molusquicidas. No obstante, a pesar de los importantes beneficios tangibles en materia de salud pública, esta integración se basa en la experiencia práctica y aún no se ha descrito lo suficiente en un contexto ecologista. Este hecho nos impide conocer hasta qué punto los diferentes componentes del plan de control integral contribuyen a interrumpir la transmisión de la enfermedad, lo que a su vez dificulta la aplicación de la misma estrategia en otros contextos. En este artículo de opinión, hemos descrito y debatido estos desafíos, así como las oportunidades y las investigaciones necesarias para avanzar.
Conclusiones: Urge formalizar un marco ecologista para el plan de control integral que permita una investigación para mejorar la comprensión mecánica, la cuantificación y la predicción de los esfuerzos de control.

Translated from English version into Spanish by Sara Solá Portillo, proofread by Lara Mattheus, through

TRANSLATORS
WITHOUT BORDERS
